# Competition for popularity and interventions on a Chinese microblogging site

**DOI:** 10.1371/journal.pone.0286093

**Published:** 2023-05-23

**Authors:** Hao Cui, János Kertész

**Affiliations:** Department of Network and Data Science, Central European University, Vienna, Austria; University of Edinburgh, UNITED KINGDOM

## Abstract

Microblogging sites are important vehicles for the users to obtain information and shape public opinion thus they are arenas of continuous competition for popularity. Most popular topics are usually indicated on ranking lists. In this study, we investigate the public attention dynamics through the Hot Search List (HSL) of the Chinese microblog Sina Weibo, where trending hashtags are ranked based on a multi-dimensional search volume index. We characterize the rank dynamics by the time spent by hashtags on the list, the time of the day they appear there, the rank diversity, and by the ranking trajectories. We show how the circadian rhythm affects the popularity of hashtags, and observe categories of their rank trajectories by a machine learning clustering algorithm. By analyzing patterns of ranking dynamics using various measures, we identify anomalies that are likely to result from the platform provider’s intervention into the ranking, including the anchoring of hashtags to certain ranks on the HSL. We propose a simple model of ranking that explains the mechanism of this anchoring effect. We found an over-representation of hashtags related to international politics at 3 out of 4 anchoring ranks on the HSL, indicating possible manipulations of public opinion.

## Introduction

Studying public attention is important from various aspects including governance, public security, marketing, and pandemic management [1, 2]. With the development of digital technology, social media have permeated into the society and become an inevitable source of information for many. An increasing part of the public obtains the latest information from social media, expresses opinions and attitudes there. This results in a strong competition for visibility and popularity partly because of their obvious relation to power and financial gain. Due to the large number of users and the volume of activity, changes in popularity happen at a high pace. As the novelty of the hot topics fades rapidly with time [3], public attention shifts to new, current trending topics. Popularity trends on social media can be regarded as a proxy of collective public attention. In the social media ecosystem, microblogging sites are important platforms because of the conciseness of the messages, the high turnover speed and the open online social networks of their users. Microblogging sites are complex interacting systems where users generate content and disseminate information through posts, reposts, comments, likes, and mentions, and where emergent phenomena occur like macroscopic collective behaviors triggered by some pieces of information [4].

Detecting and indicating the trends in popularity is important since they reflect the most concerned issues by the public in real time, which is highly relevant for a number of issues, including the popularity of cultural products, market changes [5], government policy-making, and elections [6]. In order to inform customers, microblogging site providers like Twitter or Sina Weibo present trending lists based on some statistical measures. The lists have twofold roles: First, they indicate popularity of given topics, second, they boost popularity of those topics which manage to get to the list. Therefore there is a competition in getting to the list and staying there as long as possible.

Twitter trending list shows hot topics around the globe. Research studies have analyzed the dynamics of trending topics through comprehensive statistical analysis from the aspects of lexical composition, trending time, trend re-occurrence [5], etc. There have been different factors identified, which contribute to the success of a topic, like novelty of the piece of information and the resonance level of the messages spread as well as the influence of certain members of the propagating network [7]. The evolution of Twitter trends is characterized by phases of burst, peak and fade [8] and the patterns of temporal evolution of popularity of hashtags have been ordered into six different categories [9].

The economic and political relevance of popularity of items on online media is an incentive to the service providers to intervene into the trending lists. A linear influence model [10] was introduced to capture the network effect on endogenous diffusion of hashtags on Twitter trending list and demonstrate evidences of manipulation [11] on the observed dynamics. Certain trending topics on Twitter may be opportunistically targeted with desirable qualities by spammers [12]. Recent studies on Twitter trends have found likely presence of coordinated campaigns in AstroTurf version to influence and manipulate public opinion during the COVID-19 crisis in Mexico [13].

The Chinese microblogging site Sina Weibo is geographically more limited but larger than Twitter in terms of number of daily and monthly active users [14]. Although it has generated less academic publications than Twitter, it has attracted considerable research attention due to its enormous user participation and profound role in mainland China where Twitter is blocked [15]. The Hot Search List (HSL) on Sina Weibo, with a role similar to Twitter trending list, is a major source for people from mainland China to obtain real-time information about the popularity of topics. Research on Weibo hot topics has focused on topic dynamics from the perspective of time, geography, demographics, emotion, retweeting, and correlation [16], on similarities and differences to Twitter [17, 18], emergence mechanisms [19], patterns of popularity evolution [20], prediction [21–23], social emotions and diffusion patterns [24] as well as impact of censorship [25].

The ranking of trending contents on social media changes over time, following the rise and fall of public attention dynamics: Old trends vanish and new trends emerge. The search volumes for hashtags indicate their popularity and on Sina Weibo this quantity is supposed to be one of the main underlyings for the ranking list HSL, as reflected in the name of the list.

The exposure of hashtags on the HSL has a great promotional effect thus many are keen to be on the list, resulting in strong competition and manipulation attempts. Studies reveal that hashtags from different topical categories differ in time length of prehistory (from birth till first appearance on the HSL) and the types of accounts involved in the propagation [19]. Celebrity and entertainment related hashtags are often associated with marketing accounts [26] which can be influenced by social capital [19]. Recent findings indicate possibility of algorithmic intervention [27] from the platform provider towards COVID-related hashtags on the HSL during the COVID-19 pandemic. Research indicated that human editorial decisions were involved in the curation of Weibo trending topics with the aim of increasing user engagement [28] and that Weibo actively facilitates the production and spread of online contention to attract more users through a range of recommendation mechanisms built into the platform, including the trending topic list and channels such as Sina-owned official accounts [28]. As contents on social media can influence social perception [29], studying the dynamics of both ranking position and duration of the hashtags on the HSL can deepen our understanding about the dynamics of public attention, its relation to hashtag prehistory, and reveal ways of interventions on social media platforms.

In this paper, we study the attention dynamics on Sina Weibo by digging deeper into the characteristics of the hashtags on the HSL. We describe the patterns of the ranking position and duration of hashtags, introduce ranking trajectory classification, and uncover its relationship with the prehistory and the time of the day when the hashtag first appears on the HSL. We identify anomalies which can be attributed to intervention into the ranking by the service provider and propose a model of anchoring to explain the anomalous ranking dynamics of hashtags on the HSL. We categorize the anchored hashtags based on their semantic meaning to uncover possible motivation for interference by the service provider.

## Materials and methods

### Sina Weibo data

Sina Weibo Hot Search List is a convenient tool for netizens to follow hot topics, news events and celebrity gossip, and has gone through several reforms. In 2014, a real-time Hot Search List was launched on the client with an updating frequency of once every ten minutes [30], allowing users to see the latest hot information anytime, anywhere. The updating frequency later accelerated to every minute [30] in 2017. Until 2021, there used to be one or two advertisement rank positions included in the top 50 ranks. Since 2021, Weibo went through another reform that the advertisements hashtags are randomly placed between ranks 3 and 4 or/and ranks 6 and 7, without a rank number associated in front of the advertisement, resulting in maximum 52 hashtags on the HSL, excluding the imposed positive energy recommendation position (hot search top) above all ranks which is often promotion for positive contents. Sina Weibo has also separated a new list specifically for entertainment and celebrity related hashtags, though in the normal HSL there are still entertainment related hashtags. Our data was collected from the period before the reform. Sina Weibo HSL claims to publish the ranking of the top 50 most popular hashtags based on a multi-factor index [31]. Due to its opaque way of determining and ranking hashtags on the HSL, Weibo has been target of criticism for making financial gains as the HSL serves as an advertising tool to boost popularity. Sina Weibo responded to the criticism on 23 August 2021 and released what it called the rule of capturing the “hotness” *H*_*i*_ of a hashtag *i* at a certain time [30], in the form of the following formula:

$$\begin{array}{c} H_{i}=\left(S_{i}+D_{i}+R_{i}\right)\times I_{i}, \end{array}$$ (1)

where search hotness *S*_*i*_ refers to the search volume, including manual input search and click-and-jump search, discussion hotness *D*_*i*_ is for the amount of discussion, including original posting and reposting, reading hotness *R*_*i*_ is the spreading popularity equal to the number of readings, reflecting the spread of hotspots in the Weibo system, and interaction hotness *I*_*i*_ refers to the interaction rate of hot search results page for that hashtag.

**Fig 1:**
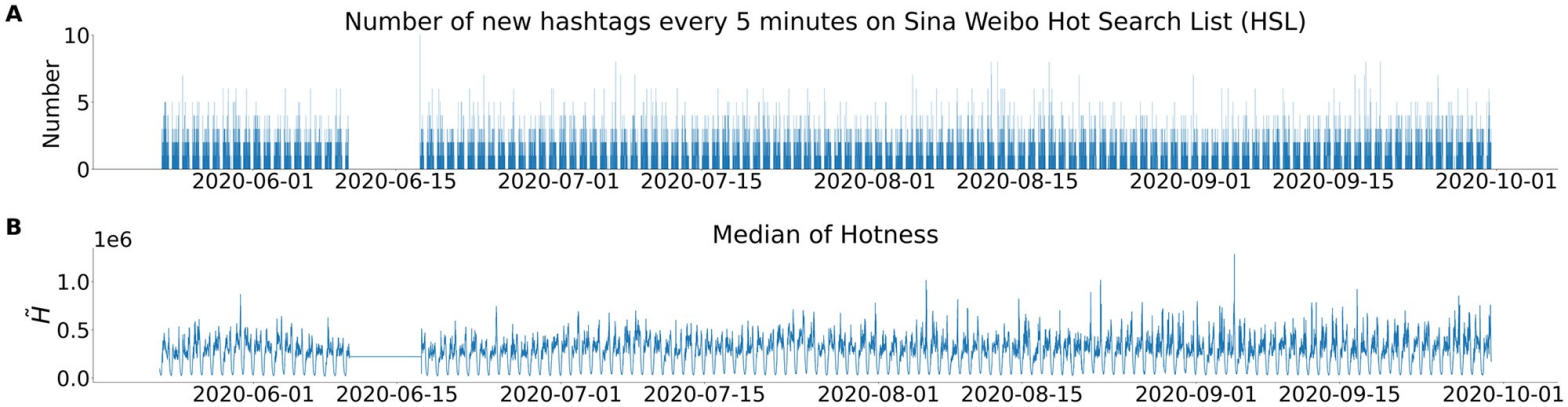
Circadian patterns of the Sina Weibo Hot Search List (HSL). **(A)** Increment of number of new hashtags per Δ*t* = 5 minutes on the HSL during the observation period from 22 May 2020 to 29 September 2020. **(B)** Time series of the median of search volume index of all hashtags on the HSL at a timestamp, advertisement rank positions excluded. $\tilde{H}$ represents the median value hotness *H* of hashtags on Sina Weibo HSL at a timestamp. In both **(A)** and **(B)** the one-week gap due to the suspension of HSL by the cyberspace authority of China is visible. An enlarged part of **(A) is in** S1 Appendix.

Sina Weibo HSL contains the names of the hashtags, their ranks and the search volume hotness which is the base of the ranking (see Eq (1)). We crawled the data from Sina Weibo HSL, with a frequency of Δ*t* = 5 minutes from 22 May 2020 to 29 September 2020. Since the commercial advertisements randomly occupied the HSL at the third and the sixth ranks, in order to get a constant length of non-advertisement hashtags on the HSL at each timestamp, we removed all the advertisement hashtags which are labeled with “Recommendation (荐)”, re-ranked the original HSL and took the top *L* = 48 hashtags for each timestamp, with *L* being the length of the list. Weibo was punished by the cyberspace authority of China to suspend the update of HSL for one week in June 2020 due to its interference with online communication [32], which causes a gap in the data (see Fig 1). We then did our major analysis based on the data after the punishment. We took all the hashtags that have appeared on the HSL in a two month period from 17 July to 17 September 2020, and crawled all the posts containing these hashtags in their prehistory from birth till first appearance on the HSL. The datasets used in this research are available in a GitHub repository [33]. The collection and analysis methods comply with the terms and conditions for the source of the data.

### Ranking dynamics

#### Measures

A popular hashtag *i* enters the HSL at time *t*_*i*_ at enter-rank *r*_*i*_(*t*_*i*_) with 1 ≤ *r*_*i*_(*t*_*i*_) ≤ *L* and disappears from it at time *T*_*i*_ at leave-rank *r*_*i*_(*T*_*i*_). During the period *t*_*i*_ ≤ *t* ≤ *T*_*i*_ the rank of this hashtag changes with time producing a trajectory *r*_*i*_(*t*) on the HSL. In order to capture the ranking characteristics of hashtags at different ranks, we use the measure rank diversity [34, 35]. Rank diversity *D*(*k*) measures the number of different hashtags at rank 1 ≤ *k* ≤ *L* during a given period of time *t*_min_ ≤ *t* ≤ *t*_max_:

$$\begin{array}{c} D\left(k\right)=\sum_{t}\sum_{i}\delta \left(k,r_{i}\left(t\right)\right)\phi_{i,k}\left(t\right), \end{array}$$ (2)

where *δ*(⋅, ⋅) is the Kronecker delta and *ϕ*_*i*_(*t*) is the indicator, which is 1 if hashtag *i* has not been at rank k until time *t* and 0 otherwise.

Rank diversity has been studied extensively. It is known that these quantities are characterized by profiles: For high ranks, their diversity have small values, while the behavior for lower ranks depends on whether the system is closed (only the rank changes but the items do not) or open (when items arrive on and leave from the list). In closed systems the dynamics at low ranks is also suppressed leading to low values of *D* and a maximum at intermediate ranks, while in open systems these quantities grow monotonously, as it can be shown by simple diffusive models [34–36]. An open system can be considered as a part of a very large closed system.

The duration *d*_*i*_ of a popular hashtag on the HSL measures the time over which it is able to attract consistent public attention:

$$\begin{array}{c} d_{i}=T_{i}-t_{i}. \end{array}$$ (3)

Note, that “high rank” means small rank value, i.e., the highest rank has rank 1. The highest rank $r_{i}^{\text{min}}$ of a hashtag measures its maximum relative ability to attract public attention during its whole lifetime on the HSL:

$$\begin{array}{c} r_{i}^{\text{min}}=\underset{t\in [t_{i},T_{i}]}{\text{min}}\;r_{i}\left(t\right). \end{array}$$ (4)

#### Categorization of rank trajectories

The rank trajectory *r*_*i*_(*t*) is uniquely defined for ∀ hashtag *i*. Some hashtags have short lifetime on the HSL, others can attract popularity for a longer period of time; some go rapidly to high ranks, others never reach that level. Are there similarities between different shapes of the trajectories and can they be ordered into categories? Here we use machine learning techniques to find characteristic patterns in these rank trajectories. In order to deal with rank time series of different lengths, we use Dynamic Time Warping (DTW) [37] as a similarity measure between two time series. DTW computes the best possible alignment between two time series. Then we use k-means clustering to find clusters of characteristic shapes. The computation was done using python tslearn package [38].

## Results

### Circadian patterns

Human actions are largely influenced by the circadian rhythm and so are online activities. Fig 1A shows the increment of the number of hashtags per Δ*t* = 5 minutes interval clearly demonstrating the cyclic structure during the observation period from 22 May 2020 to 29 September 2020, except for a short interruption in June 2020. Similarly in Fig 1B, the median search volume index of hashtags on the HSL at a timestamp rises and decays in a periodic fashion. The missing of data for one-week in June 2020 is observed in both Fig 1A and 1B, which results from the suspension of HSL by the cyberspace authority of China due to Weibo’s interference with online communication [32].

### Rank trajectory clustering

A successful hashtag *i* stays on the HSL between the time instants *t*_*i*_, when it appears on the list, until *T*_*i*_, when it finally disappears from it defining the duration *d*_*i*_ = *T*_*i*_ − *t*_*i*_. Some hashtags stay on the list for very short time (*d*_*i*_ < 10 minutes), while some others stay for many hours. The rank of a hashtag *i* follows a trajectory *r*_*i*_(*t*). Some hashtags’ trajectories first go to higher ranks (smaller rank numbers) and then drop, some go higher and lower and higher again, there are also cases that hashtag’s trajectory goes higher and then it disappears. Also, the speed change of the trajectories is variable, resulting in a multitude of shapes of rank trajectories.

**Fig 2:**
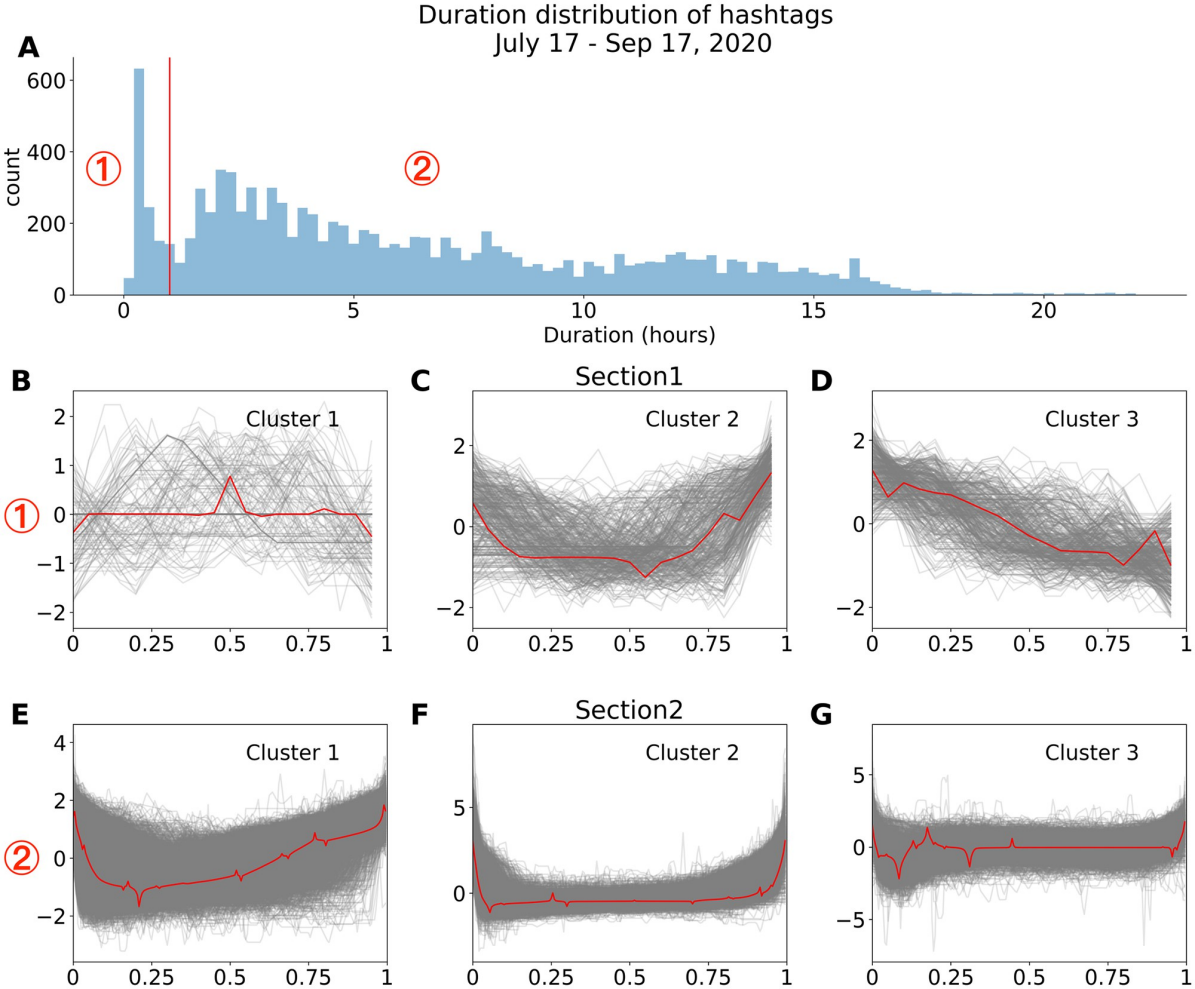
Clustering patterns of hashtag rank trajectories on the Sina Weibo HSL. **(A)** Distribution of hashtag duration on the HSL, divided into two sections based on local minima at 1 hour. Results of k-means clustering with 3 clusters in each section for time series data are shown, metric is Dynamic Time Warping (DTW) distance, y-axis is normalized to the mean and the standard deviation and the x-axis by *d*_*i*_. **(B)**, **(C)**, **(D)** correspond to duration interval from 0 to 1 hour (Section 1). **(E)**, **(F)**, **(G)** correspond to duration interval larger than 1 hour (Section 2). Red curves depict clustering centers (centroid) [39], computed as the barycenters [40] with respect to DTW. (We performed the clustering also with 4 clusters for both categories, see S1 Appendix).

The duration distribution of hashtags in the observation period is shown in Fig 2A. We observe a sharp peak for hashtags with short duration and two less pronounced peaks. The vertical red line at the local minimum of 1 hour separates the duration distribution into two sections, section 1 and 2, respectively. The individual rows in Fig 2 correspond to the clustering of the rank trajectories on each of the separated sections: Section 1 (B,C,D) and Section 2 (E,F,G). Even for hashtags with short duration on the HSL (Section 1) it is worth categorizing the rank trajectories. In most cases the rank does not change much during the lifetime *d*_*i*_ (see Fig 2B and 2C) and remains at low ranks (large rank numbers), however, as shown in Fig 2D, some ranks of the hashtags exhibit a clear directional motion: they go to higher ranks and disappear from there. For the more expected rank trajectories shown from Fig 2E to 2G, we also see some recognizable differences. Rank trajectories in Fig 2E first go to higher ranks and quickly go to lower ranks after hitting the top, without staying at a certain rank for a long time. Rank trajectories in Fig 2F first go higher, stay stable around the highest ranks with little fluctuation for a long time and then go down. Rank trajectories in Fig 2G first go higher, with more fluctuations but never surpass the previous peak, then stay stable for a long time and finally go down the ranks. In the next Section we will show how the rank trajectory shapes are related to the time of the day the hashtags first appear on the HSL.

### Duration

**Fig 3:**
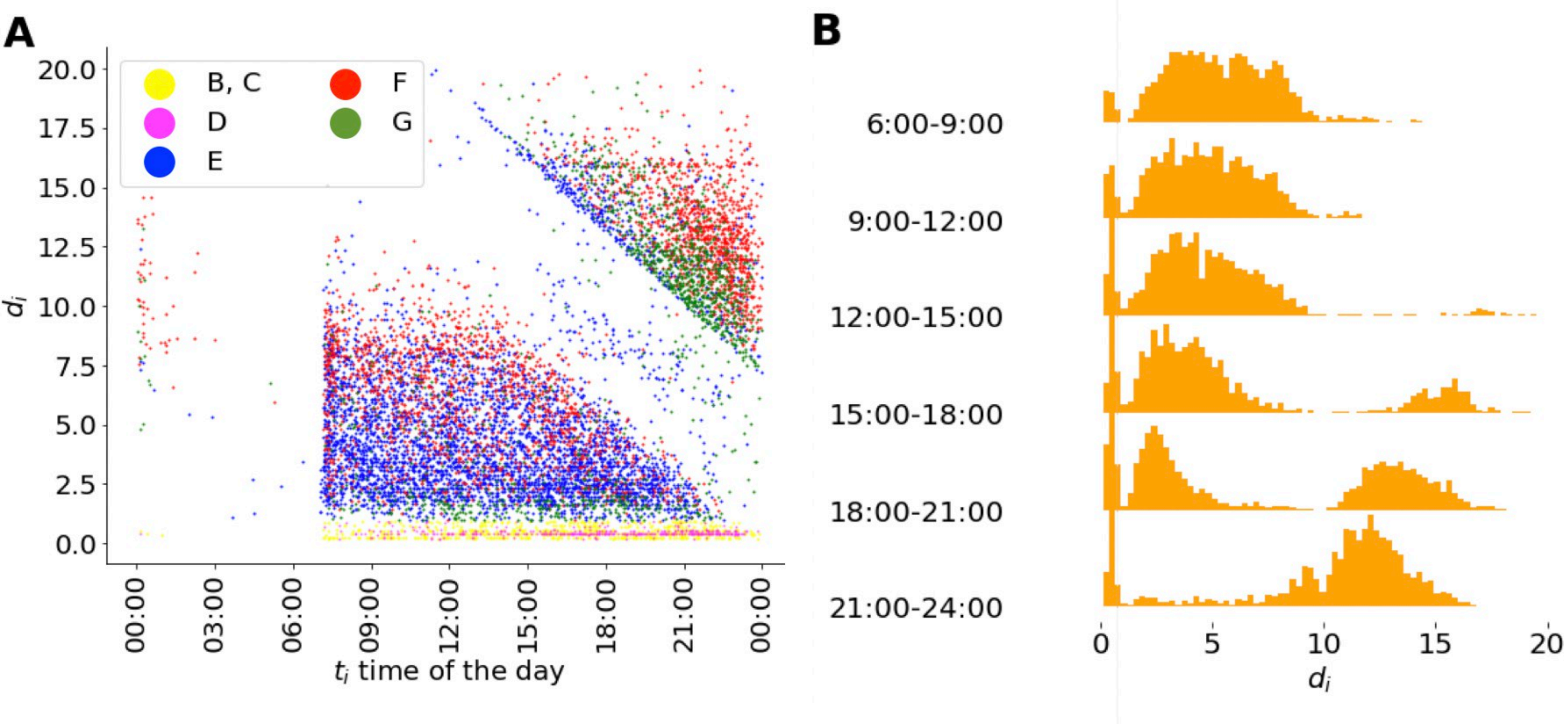
Relationship between hashtags’ duration on the HSL and the time *t*_*i*_. **(A)** Scatter plot of hashtags’ duration on the HSL and the time of the day they first appear on the HSL. Each point is a hashtag, colored by the category it is clustered in Fig 2. **(B)** Distribution of hashtags’ duration on the HSL according to different time intervals during the day of first appearance on HSL.

Fig 3A is the *d*_*i*_ vs *t*_*i*_(mod 24h) scatter plot, i.e., it shows the durations of the hashtags vs the times of the day when they first appeared on the HSL, with each point representing a hashtag. Hashtags tend to appear on the HSL starting from around 7 a.m. till midnight. We can see clear shapes of lower-left and upper-right triangles, separated by a stripe in the middle with a low number of points inside. The lower boundary of the upper-right triangle is very sharp, while the upper boundary of the lower-left triangle is less so. There are data points within the stripe, but the density is much less compared with the data points inside the triangles and also if we compare it to the users’ overall activity pattern (see S1 Appendix). The vertical distance between the triangle boundary lines is approximately 7 hours. The existence of these triangles suggests that the hashtags, which enter the HSL after 15 p.m. tend to either disappear from the HSL on the same day or stay on the HSL during the night and disappear after 7 a.m. the next day. This is presumably related to Sina Weibo working mode, already pointed out in previous studies [19], namely that Sina Weibo practically stops putting new hashtags onto HSL between midnight and 7 a.m. If the ranking was automated following the formula Eq 1, the changes from day to night should not be that sharp and the circadian pattern should follow more or less that of the people’s activity.

Fig 3B shows the duration distribution of the hashtags as a decomposition of Fig 2A by binned starting values of the times of the day. For each time interval, the observed distribution is trimodal. As the start time of the day *t*_*i*_(mod 24h) goes on, the density of hashtags in the third mode is increasing. In Fig 3A we see a low-density area at around 1 hour duration between the blue and the yellow dots, which corresponds to the minimum between sections 1 and 2 in Fig 2A. Accordingly, in the duration distribution plot shown in Fig 3B, a peak is observed for hashtags with duration shorter than 1 hour. Within this stripe in Fig 3A, there is an accumulation of pink dots corresponding to trajectories of category D, with a unique shape, namely starting at low rank and ending at a high one within a short period of time. In most other cases the more expected shapes are observed, namely starting and ending from low ranks and having in between some higher ranks. Simple categorization of hashtags from each of the clusters based on semantic meaning does not point toward the relationship between the rank trajectories and the contents of the hashtag, as shown in Table S1 in S1 Appendix. Later we will show that such an analysis for specific, anchoring ranks reveal systematic deviations from average behavior.

How are the shapes of the rank trajectories related to the time of the day the hashtags first appear on the HSL? Recall the Weibo working mode, if a hashtag’s stay on the HSL is influenced by the night break, then it will automatically have a little-fluctuation period of at least seven hours, resulting in a rank trajectory shape similar to Fig 2F or the last part of Fig 2G, which we color in red and green respectively in Fig 3A. Hashtags in Fig 2F are born closer to midnight and further away from the hypotenuse of the upper-right triangle in Fig 3A. This is reasonable since hashtags entering HSL close to midnight are likely exposed to the stay on the HSL during the night break. Hashtags with shape in Fig 2G, however, are close to the hypotenuse boundary of the upper-right triangle in Fig 3A. One possible explanation is that although these hashtags’ attention level is already in decreasing trend, their stay on the HSL are prolonged by the night break, so that when the next day begins, they are replaced by new hashtags and leave the list. The majority of hashtags with shape shown in Fig 2E are of shorter duration, located in the dense area of the lower-left triangle colored in blue in Fig 3A. The separation of the red and blue areas in Fig 3A lower-left triangle tells that hashtags which quickly go down after reaching their highest ranks on the HSL lack the ability to consistently attract public attention to maintain their positions on the list. In contrast, hashtags maintain relatively stable ranks (Fig 2F) stay longer times on the HSL, as Fig 3A lower-left red area suggests.

### Ranking

**Fig 4:**
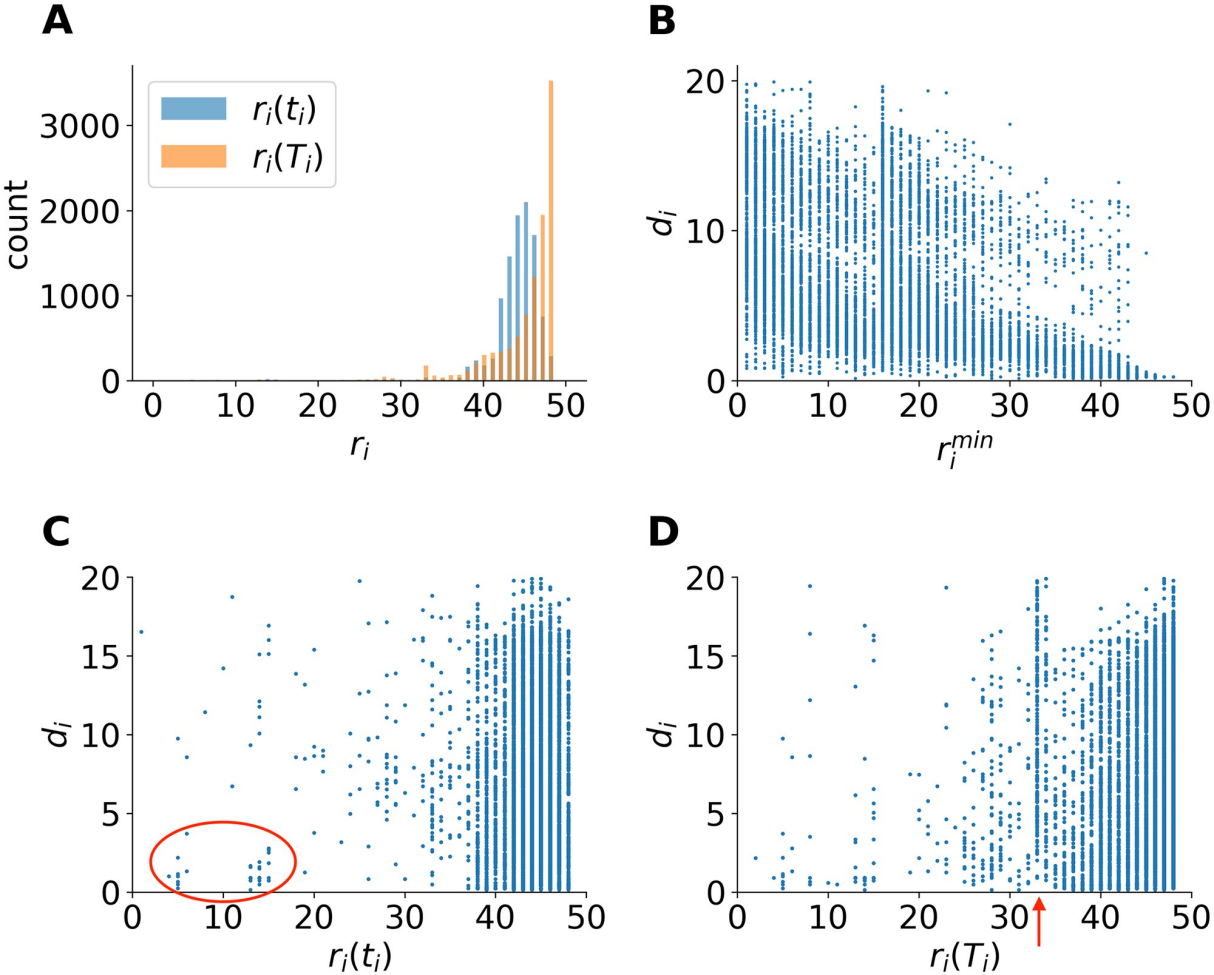
Ranking dynamics characterization of hashtags on the Sina Weibo HSL from 17 July 2020 to 17 Sep 2020. **(A)** Distribution of *r*_*i*_(*t*_*i*_) and *r*_*i*_(*T*_*i*_). **(B)** Scatter plot of $r_{i}^{min}$ and *d*_*i*_. **(C)** Scatter plot of *r*_*i*_(*t*_*i*_) and *d*_*i*_, hashtags with high enter-rank and short duration are circled red. **(D)** Scatter plot of *r*_*i*_(*T*_*i*_) and *d*_*i*_, rank 33 marked by red arrow.

The popularity of a hashtag is reflected in its rank position and the duration it stays on the HSL. Fig 4A shows the distribution of the enter-ranks *r*_*i*_(*t*_*i*_) and leave-ranks *r*_*i*_(*T*_*i*_) on the HSL. The majority of hashtags do not land on the HSL from the very bottom of the ranking list, instead they tend to enter at ranks 44–46 while they tend to leave from the bottom ranks. Fig 4B shows the scatter plot of the highest rank of the hashtags and their duration on the HSL. The duration exhibits a bimodal pattern with a sudden jump at rank 16, and then it decreases. Fig 4C shows the relationship between the hashtags’ enter-ranks on the HSL and their corresponding duration on the HSL. As known from ranking dynamics [35], items at high ranks stay longer, thus hashtags at higher ranks are more stable and stay for longer hours on the HSL, so that it is strange for hashtags entering HSL at a high rank and only stay for a short duration, as marked in the red circle in Fig 4C. We found those hashtags are mostly related to celebrities, games, or TV programs, see S1 Appendix. In terms of the leave-rank and duration as shown in Fig 4D, rank 33 show a clear statistical deviation from the rest of the ranks, as marked by a red arrow. Surprisingly, we found the majority of those hashtags that leave HSL at rank 33 are related to international politics, to name a few, #UK suspends extradition treaty with Hong Kong# (#英国暂停与香港间的引渡条约#), #The United States announced sanctions against 24 Chinese companies involved in the construction of islands in the South China Sea# (#美宣布制裁24家参与南海建岛中企#), #Russian foreign minister says won’t reject 5G cooperation with China# (#俄外长称不会拒绝与中国开展5G合作#). See S1 Appendix for the whole list of hashtags with leave-rank 33 together with their translation and examples of rank trajectories.

**Fig 5:**
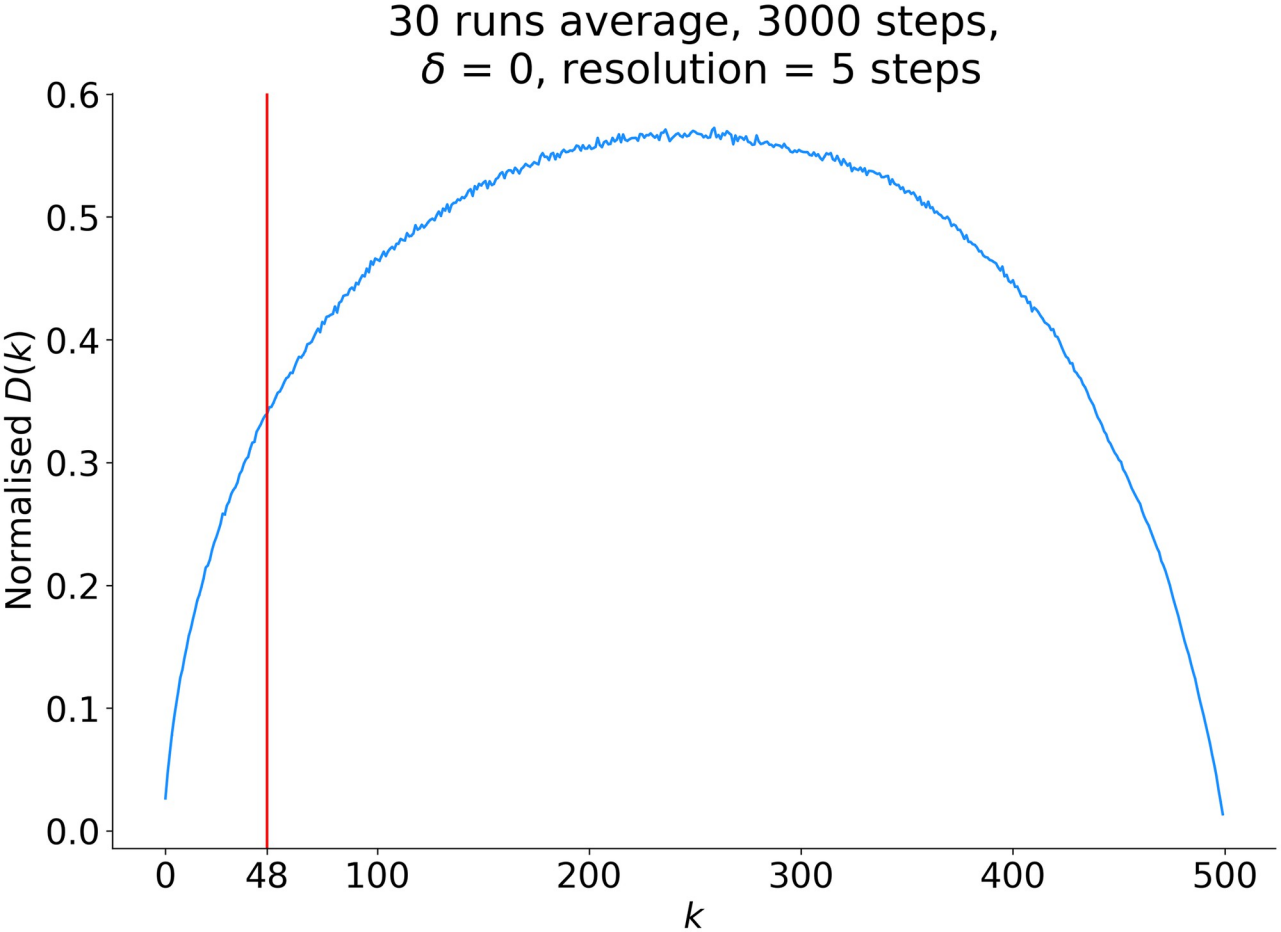
Parabola shaped normalized rank diversity in a model closed system of size 500. The top L = 48 can be considered as an open system.

As mentioned in the Introduction, spontaneously evolving ranking dynamics have typical rank diversity patterns [34–36]. A simple diffusional ranking model shows them clearly. Let us take a system of *N* elements (for the hashtags), each element has a random initial score of values within (0, 1), and rank these elements from top to bottom based on the scores. Let *r*_*i*_ and *s*_*i*_ denote the rank and the score of the *i*-th element, respectively. The scores change in time and that causes the rank movement of the elements. An element is randomly selected, 1 is added to its score and the ranking is changed if necessary. After sufficiently averaging the rank diversity, the curve shape is smoothed. Depending on whether the system is closed or open, the rank diversity curve shows a parabola shape or a portion of it, as shown in Fig 5.

### Ranking dynamics in relation to prehistory

**Fig 6:**
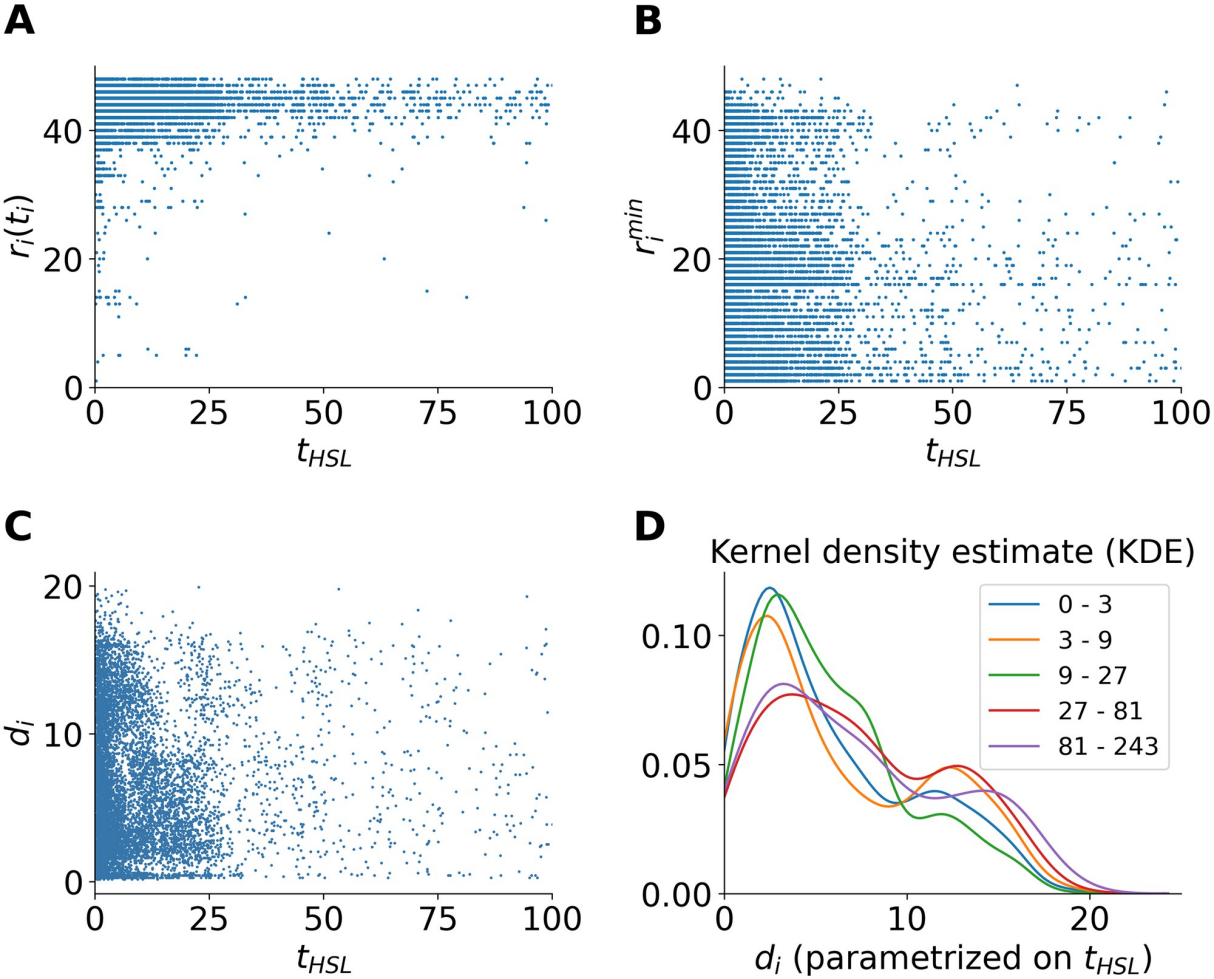
Prehistory length *t*_*HSL*_, enter-ranks *r*_*i*_(*t*_*i*_), the highest rank $r_{i}^{min}$, and duration *d*_*i*_ of hashtags on the Sina Weibo HSL. **(A)** The relationship between the hashtags’ prehistory time length and the ranks they first enter on the HSL. **(B)** The relationship between the hashtags’ prehistory time length and the highest rank during stay on the HSL. **(C)** The relationship between the hashtags’ prehistory time length and the duration they stay on the HSL. **(D)** Parameterized probability density function of the hashtag duration on the HSL by prehistory time length, using kernel density estimation (KDE) [41], with the parameter bw = “scott” [42].

Before the hashtags gain enough popularity and land on the HSL, they go through different propagation routes during their prehistory. The time length of the prehistories *t*_*HSL*_ differ for different hashtags [19]. Some hashtags get to the HSL in very short time after birth, while others take longer. Fig 6 shows the relationship between *t*_*HSL*_ of the hashtags, the ranks they enter the HSL *r*_*i*_(*t*_*i*_), the highest rank $r_{i}^{min}$, and the duration *d*_*i*_ of their stay on the HSL. As shown in Fig 6A, in accordance with Fig 4A, the majority of hashtags enter the HSL at a low rank peaking around 45. Some hashtags enter the HSL at higher ranks, however, as the prehistory gets longer, the chance the hashtag enters the HSL from a high rank is less likely. Fig 6B suggests longer prehistory means in most cases higher top ranks (*r*^*min*^ < 20). As shown in the previous work [19], the hashtags about stars are over-represented in this category. As for the properties of hashtag duration on the HSL shown in Fig 6C and 6D, the duration against prehistory length exhibits bimodal distribution. As the prehistory length increases, the first peak drops and the second peak rises. The bimodality similar to results shown in Fig 3, is influenced by the Weibo circadian working mode.

### Anchor effect

The dynamics of popularity as captured in the HSL should be sensitive to the actual trends and reflect the users’ overall activity patterns. The individual rank trajectories show fluctuations but after averaging one would expect smooth behaviors. However, when studying the characteristics of the hashtags’ rank dynamics on HSL, like the rank diversity we bumped into strange behaviors which we interpret as indications of interventions by the service provider.

Here we generalize the ranking model introduced in Section Ranking to incorprate the anchoring effect to simulate the dynamics of the hashtag ranking anomalies on the HSL. The idea of the anchor is the following: Set an anchor at position *A*. For hashtags whose *r* < *A*, it is difficult to go down the ranking list; for hashtags whose *r* > *A*, it is difficult to go to higher ranks (note that high rank means low *r* value). The anchor represents a barrier characterized by an increment *δ*. Let *ϕ*(*r*_*i*_) = *i* denote the selection of the element at a given rank at an instant of time.

The procedure of ranking at each step is shown below. Randomly pick one element *j* and $s_{j}^{\text{new}}=s_{j}+1$. There are three possibilities:

(a) *r*_*j*_ < *A*. Update the top *A* − 1 ranks, no change of the anchor element.
(b) *r*_*j*_ = *A*. If *k* = *ϕ*(*A* − 1) and $s_{j}^{\text{new}}>s_{k}+\delta$, update the top *A* rank. Otherwise, no change of ranks.
(c) *r*_*j*_ > *A*. If *ℓ* = *ϕ*(*A*) and $s_{j}^{\text{new}}>s_{\ell }+\delta$, old anchor rank drops to *A* + 1, update the top *A* + 1 ranks. Otherwise, update ranks lower than *A*, no change of the anchor element.

We simulate a system with 500 elements and take the top *L* = 48 ranks to approximate an open system.

**Fig 7:**
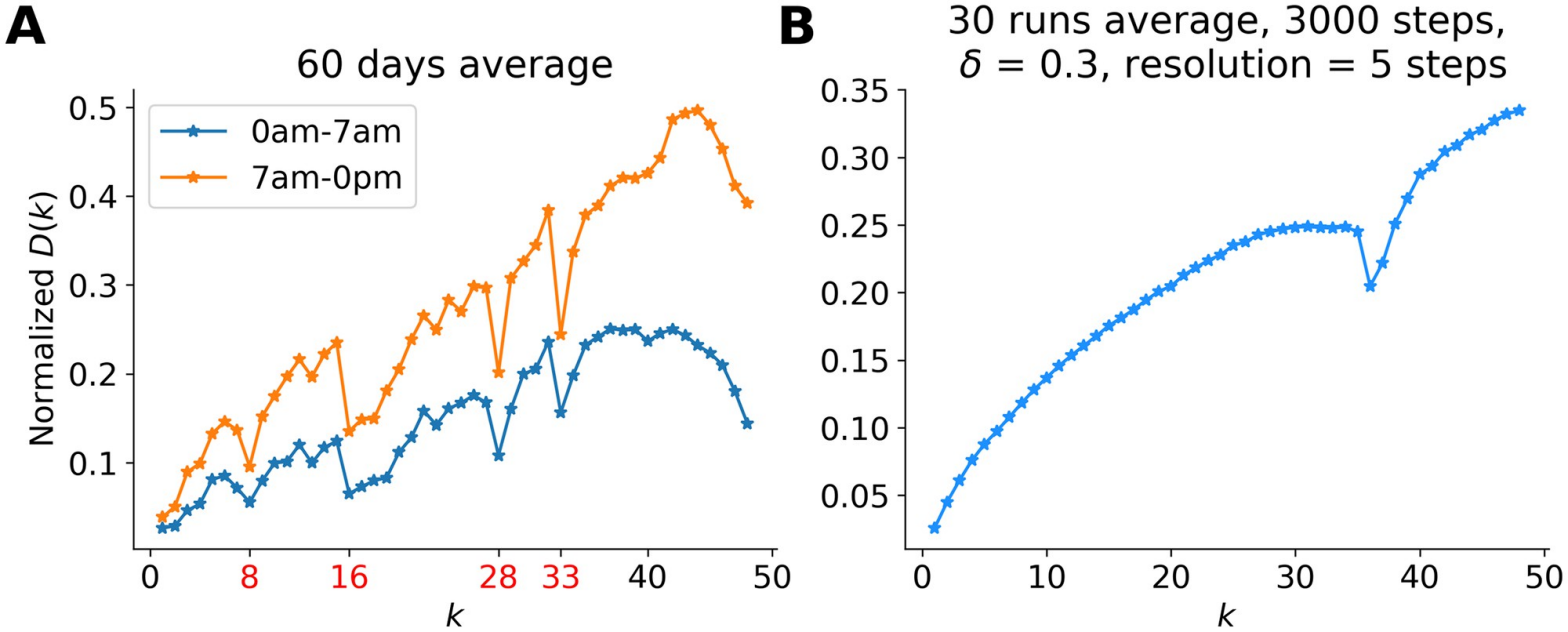
Rank dynamics comparison between empirical data and a ranking model with anchoring. **(A)** Empirical rank diversity separated for day (upper line) and night (lower line). The sudden drops are at ranks 8, 16, 28, and 33. **(B)** Simulated rank diversity with the anchor effect.

The rank diversity of a non-intervened system has parabola-shape, see Fig 5. The intervention produces a deep valley at the anchoring position, very similar to those observed in the measured curves in Fig 7 which shows the comparison between the real data and our model with anchoring. The difference between the behavior during the night and day is apparent: The former is more likely to the closed systems’ characteristics with reduced activity while the latter is closer to the open systems’ features although the trend around rank 44 turns down, probably due to the fact that the hashtags’ enter-ranks *r*_*i*_(*t*_*i*_) is shifted to the left as shown in Fig 4A. At certain positions (ranks 8, 16, 28, and 33) there are large drops in the values of the function, indicating intervention by “anchoring” hashtags at these specific ranks. With the simple model, reproducing qualitatively the effect, we support the assumption that the observed anomalies in the ranking functions are due to intervention.

**Fig 8:**
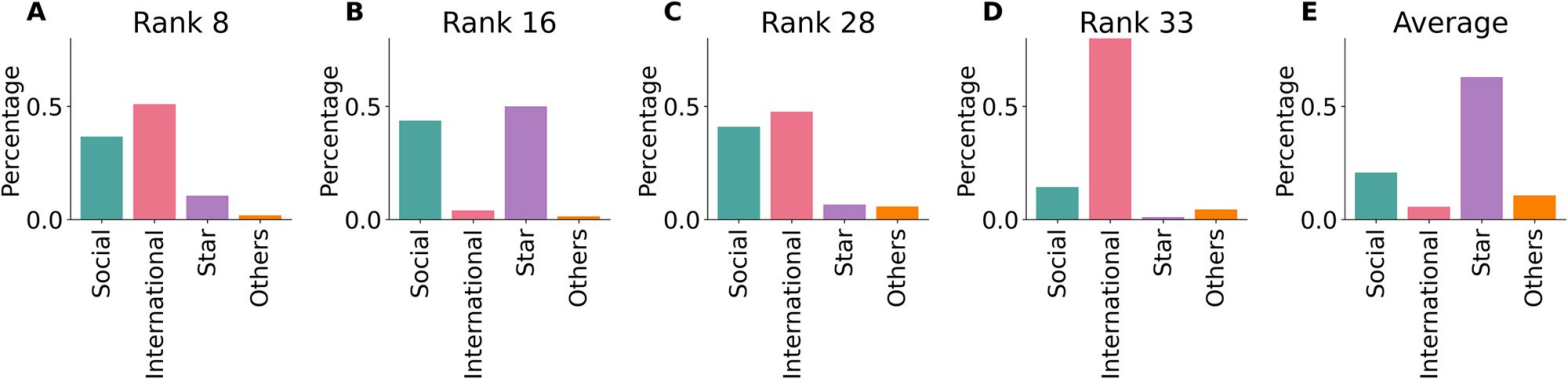
Categorized proportion of hashtags that have stayed at certain ranks on HSL for longer than 2 hours. **(A)(B)(C)(D)** show the content distribution of hashtags at ranks 8, 16, 28, and 33 respectively, corresponding to the sudden drops in Fig 7A. **(E)** Averaged proportion of hashtags by content category at ranks 5, 12, 21, 25, 30, 37.

To understand the background why some hashtags get anchored, we classified the hashtags that have stayed at the anchoring ranks for longer than 2 hours into four categories based on semantic meaning (see SI3 in S1 Appendix). Fig 8A–8D show the proportion of such hashtags by category at each of the anchoring ranks 8, 16, 28, and 33, respectively. Comparing with Fig 8E, where the percentages are the average of each categories at six non-anchoring ranks (5, 12, 21, 25, 30, 37), ranks 8 (Fig 8A), 28 (Fig 8C), and 33 (Fig 8D) clearly have a large promoted proportion of International hashtags where the majority are related to international politics. Social hashtags also have a larger proportion at anchoring ranks except for rank 33.

## Discussion

Public attention is precious and it is nowadays largely dependent on online social media, therefore it is of great interest to understand the dynamics governing popularity on such platforms. Considerable effort has been devoted to this task on Twitter [3–5, 8] and some results are also known on Sina Weibo [16, 17, 19]. In order to attract attention, people, companies, and political actors are tempted to make use of hidden manipulations besides well known tools of direct advertisements or propaganda [11, 12, 15, 19, 27]. Thus popularity can emerge spontaneously via collective attention from online users who are genuinely interested in a topic and form trends, quantified and captured by the algorithm of the platform, or trends emerge from intervention by the platform provider motivated by financial or other interests. (It should be noted that “collective attention” may also be influenced, e.g., by spamming [12] or coordinated campaigns [13, 17]).

In this paper, we studied the attention dynamics of trending hashtags on the Sina Weibo Hot Search List by using various measures of ranking dynamics, like entering and leaving ranks as well as duration of hashtags on HSL, rank diversity, and categories of rank trajectories. The aim of the identification of regularities in the ranking dynamics was twofold: First, contribution to the quantitative characterization of the dynamics of public attention in order to better understand its mechanism, and second, finding signatures of possible interventions by the service provider.

The duration of the hashtags on the HSL in relation to the time of the day they enter the list shows trimodality (Fig 3). This is related to the fact that the appearance of hashtags on the HSL have circadian patterns (Fig 1A). On the one hand, the pattern is caused by the circadian rhythm of the users whose activities depend on the time of the day (see S1 Appendix), on the other hand it is imposed by the apparent working mode of Sina Weibo, which reduces the night-time flow of new hashtags to the HSL almost to zero level. The night break is reflected in the very low number of points in the stripe separating the two triangles in Fig 3A and in the particularly sharp upper boundary of this stripe. This seven hour gap has been shown to influence the prehistory of the successful hashtags [19] by contributing to the difference between shorter and longer prehistories and it creates a link between the behavior of the hashtags on the HSL and their prehistory (Fig 6).

The distinction between user daily posts volume shown in Fig S1 in S1 Appendix and the sharp day-night boundaries of daily patterns in Fig S2 in S1 Appendix is already an example that we are able to identify interventions by the service provider, that the ranking is not automated following a plain formula like Eq 1 but depends on human control. More importantly, we show an anchoring effect at some rank positions on the HSL, where rank diversity is suppressed as compared to the expected smooth behavior of this quantity. Using a simple ranking model we show how anchoring at some rank positions changes rank diversity. A further observation indicating intervention is that some hashtags on the HSL appear at high ranks and disappear in short time (Fig 4C), we found these hashtags are mostly from the Star category, (see S1 Appendix for the categorization and the list of the hashtags). Similarly, there are many hashtags that just stay on the HSL for short time which is shown in the first peak in Fig 3B. The fact that the peak is separated from the rest of the distribution is also likely be related to intervention.

Our method cannot tell the origin of the interventions, whether they result directly from Weibo, pollution by bots, or internet water armies [43] that could blur the picture of the natural activities originating from normal users. The fact that the irregularities occur at specific ranks, which would be difficult to target by external influence makes the intervention by the service provider more likely. The reason or motivation for the possible intervention is unknown, we can only make reasonable guesses based on statistical analysis, whether it is due to the government’s “promote positive contents” campaign, or the influence of social capital to promote certain advertisements, etc. For example, the proportion of International hashtags that have stayed for longer than 2 hours at the anchoring ranks (8, 28, 33) are much larger than the averaged value of several other non-anchoring ranks. In addition, we found that the hashtags leaving the HSL at rank 33 are mostly related to international news, predominantly of political nature. Moreover, politics related hashtags tend to have less fluctuations than non-politics hashtags (see Fig S10 in S1 Appendix), implying possible political motivation. Ranks 8, 16, and 28 also see a higher proportion of anchored Social hashtags, this might be an implication of Weibo’s social responsibility as an important news source of social issues for the public.

Sina Weibo is the microblogging site with world-wide the largest number of active users, who are overwhelmingly Chinese speakers. While we believe that alone the size of Sina Weibo justifies focused study, we know that most of our results are idiosyncratic. However, this is true only in a narrow sense as our results provide general lessons. We demonstrated that studying the ranking dynamics in popularity lists is worth for several reasons. First, we uncovered relationships between ranking dynamics and the circadian pattern of user activity, also establishing a link to the prehistory of items getting to the ranking list. Moreover, we identified different trajectory categories on the list, which characterize different dynamic patterns of popularity. Finally, and most importantly, we showed, how pinpointing anomalies in ranking statistics can be used to identify interventions by the service provider. As service providers have financial interests and may be under political pressure, objectivity of the ranking lists and its truth content can be questioned.

As the platform algorithms may change from time to time, it is challenging to keep track of the interventions, as they can be detrimental being a possible tool of online mass manipulation. Thus, similar to the fight against fake news, the fight against manipulation of public attention is in the interest of the society and it also needs the tools of detecting interventions. Our studies give important reference not only in terms of intervention detection on social media, but also for other research disciplines, such as communication science, journalism, political science, to investigate in further details the specific messages and different aspects of online political contents, and learn more about the motivations of such interventions.

## Supporting information

S1 Appendix Supplementary material to the manuscript. (PDF)
