# Supplementary material for: Competition for popularity and interventions on a Chinese microblogging site
Source: PLoS One. 2023 May 23;18(5):e0286093. doi: 10.1371/journal.pone.0286093 (PMC10204960; doi:10.1371/journal.pone.0286093)
Supplement: S1 Appendix — (PDF) [file pone.0286093.s001.pdf]

Supporting Information to:

Competition for popularity and interventions on a Chinese microblogging site

Hao Cui[1] and János Kertész[1*]
[1]Department of Network and Data Science, Central European University, Quellenstrasse 51, A-1100 Vienna, Austria
*Correspondence: kerteszj@ceu.edu

SI1. Daily posts volume on Weibo

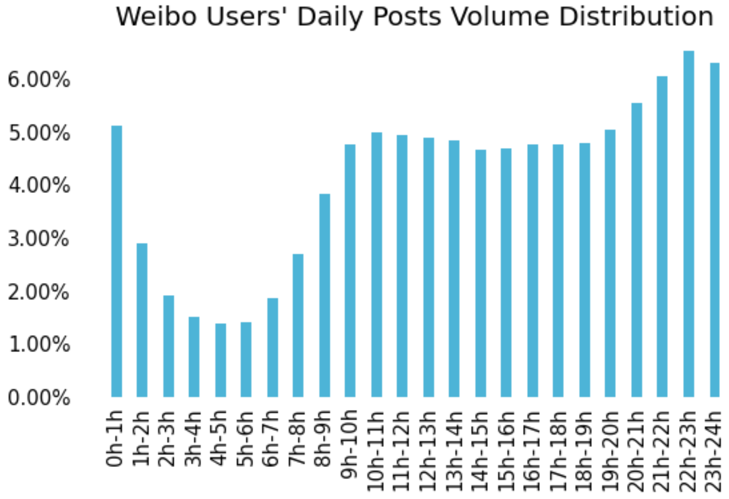

Figure S1. Distribution of Weibo users' daily posts volume according to Weibo User Development Report [1].

SI2. Enlarged part of Figure 1A

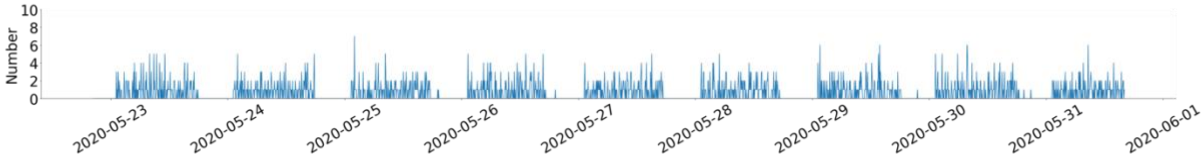

Figure S2. Number of new hashtags every 5 minutes on Sina Weibo Hot Search List (HSL). An enlarged part of Fig. 1A with clear cyclic structure.

SI3. Categorization of hashtags in each cluster and examples of rank trajectories

Table S1. Number of hashtags by category in sampled clusters of size 100

	Stars	Social	International	Others
Section 1 Cluster 1	34	46	12	8
Section 1 Cluster 2	43	35	8	14

Section 1 Cluster 3	47	36	11	6
Section 2 Cluster 1	42	40	12	6
Section 2 Cluster 2	57	28	8	7
Section 2 Cluster 3	44	30	12	14

We took random samples of size 100 for each of the clusters and classified the hashtags into 4 categories based on their semantic meaning by human judgement: Stars, Social, International, and Others. The Stars category consists of movie/sports stars, singers, idols, celebrities as well as the TV programs/movies and events that they participate in. The Social category consists of hashtags that are related to social accidents, crimes, natural disasters, and other events that are related to social life. The International category consists of hashtags whose content is related to news of regions and countries outside of mainland China. The Others category consists of the rest of the hashtags that fall into none of the above categories. There are not huge differences between the proportions of each category in each cluster. The Star category and the Social category occupies around 80% of the total sample. The following figures show the rank trajectory examples of hashtags in each cluster. The random samples of each cluster are available in the GitHub repository [2].

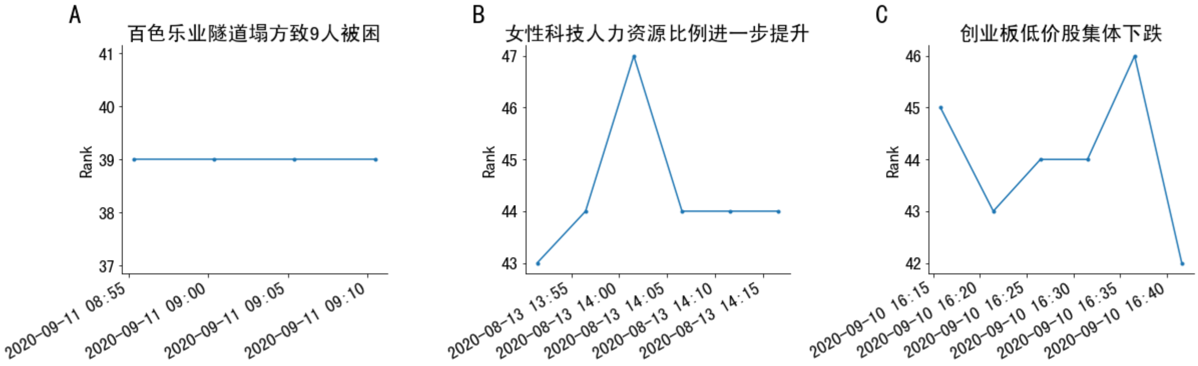

Figure S3. Examples of rank trajectories in Section 1 Cluster 1. (A) # Baise Leye tunnel collapse caused 9 people trapped# (#百色乐业隧道塌方致 9 人被困#) (B) (#The proportion of female science and technology human resources to further improve#) #女性科技人力资源比例进一步提升# (C) # GEM low-priced stocks fell collectively# (#创业板低价股集体下跌#)

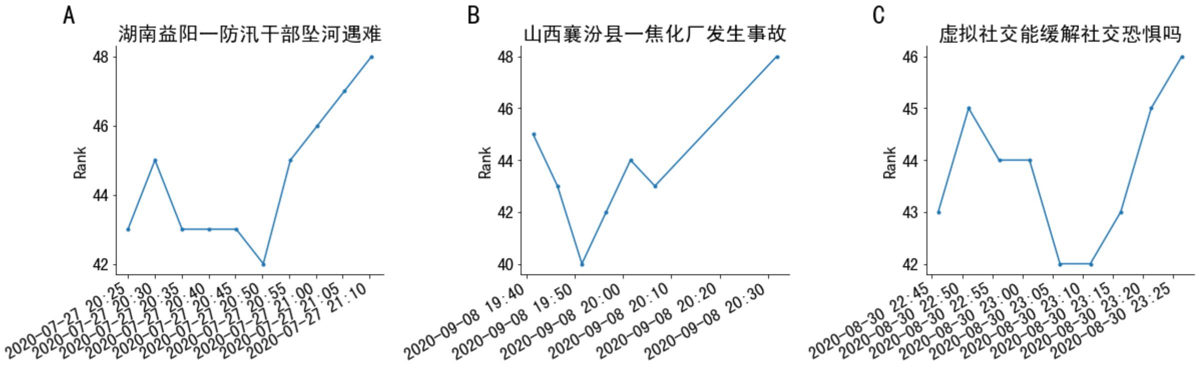

Figure S4. Examples of rank trajectories in Section 1 Cluster 2. (A) #Hunan Yiyang a flood control cadres fell into the river and died# (#湖南益阳一防汛干部坠河遇难#) (B) (#An accident occurred in a coking plant in Xiangfen County, Shanxi#) #山西襄汾县一焦化厂发生事故# (C) #Can virtual socializing ease social fears# (#虚拟社交能缓解社交恐惧吗#)

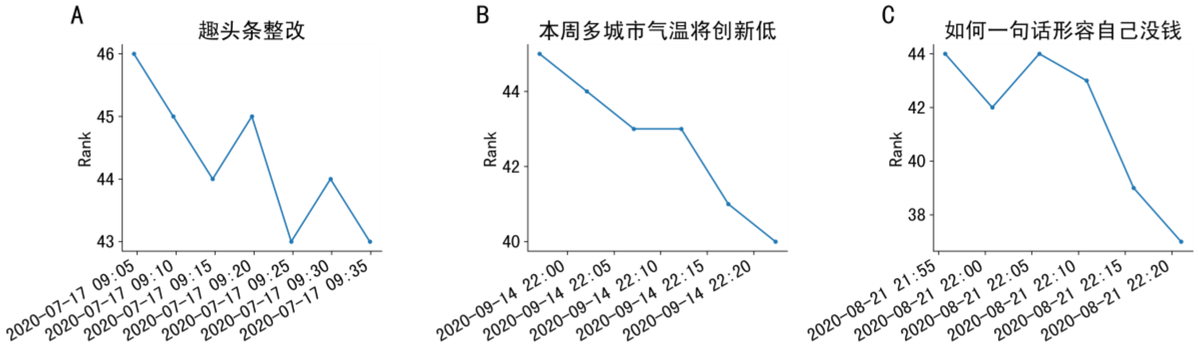

Figure S5. Examples of rank trajectories in Section 1 Cluster 3. (A) #Qutoutiao rectification# (#趣头条整改#) (B) (#Temperatures set to hit record lows in many cities this week#) #本周多城市气温将创新低# (C) #How to describe yourself having no money in one sentence# (#如何一句话形容自己没钱#)

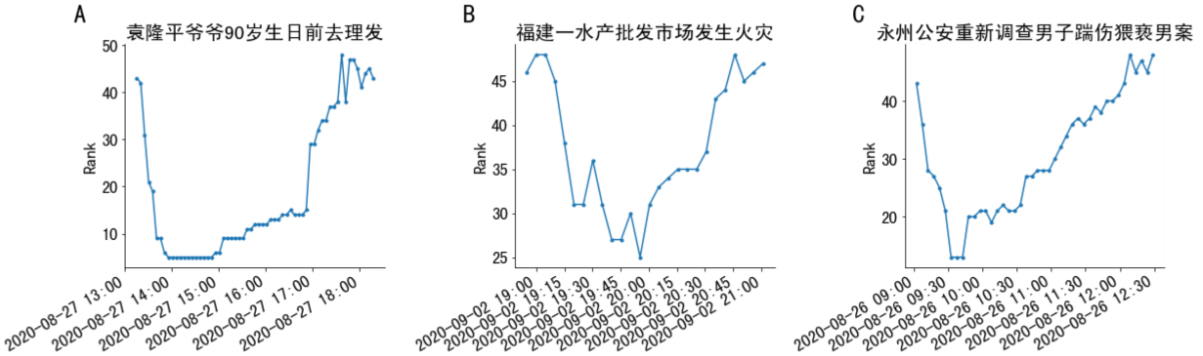

Figure S6. Examples of rank trajectories in Section 2 Cluster 1. (A) # Grandpa Yuan Longping went for a haircut before his 90th birthday# (#袁隆平爷爷 90 岁生日前去理发#) (B) (#Fire in a wholesale fish market in Fujian#) #福建一水产批发市场发生火灾# (C) # Yongzhou public security re-investigation of the man kicked and injured molesting man# (#永州公安重新调查男子踹伤猥亵男案#)

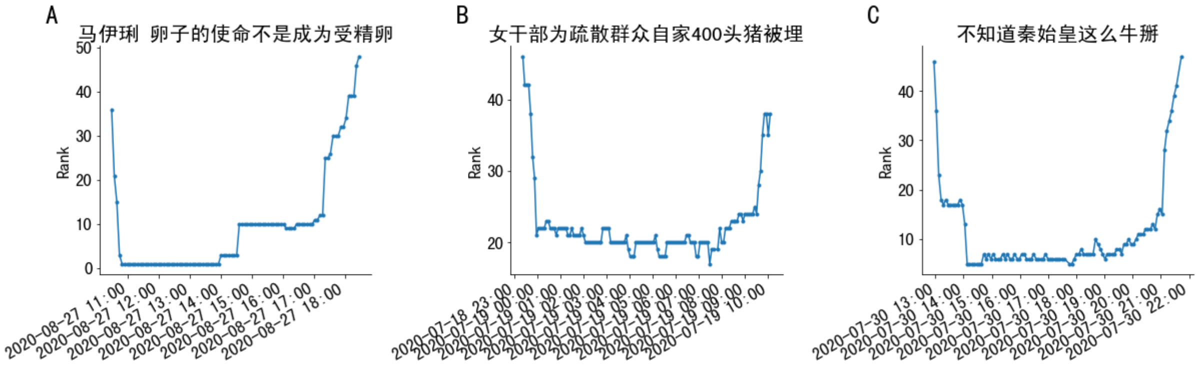

Figure S7. Examples of rank trajectories in Section 2 Cluster 2. (A) # Ma Yili The mission of the egg is not to become a fertilized egg# (#马伊琍 卵子的使命不是成为受精卵#) (B) (#Female cadres to evacuate the crowd, 400 pigs buried in own home#) #女干部为疏散群众自家 400 头猪被埋# (C) #Did not know Qin Shi Huang was so powerful# (#不知道秦始皇这么牛掰#)

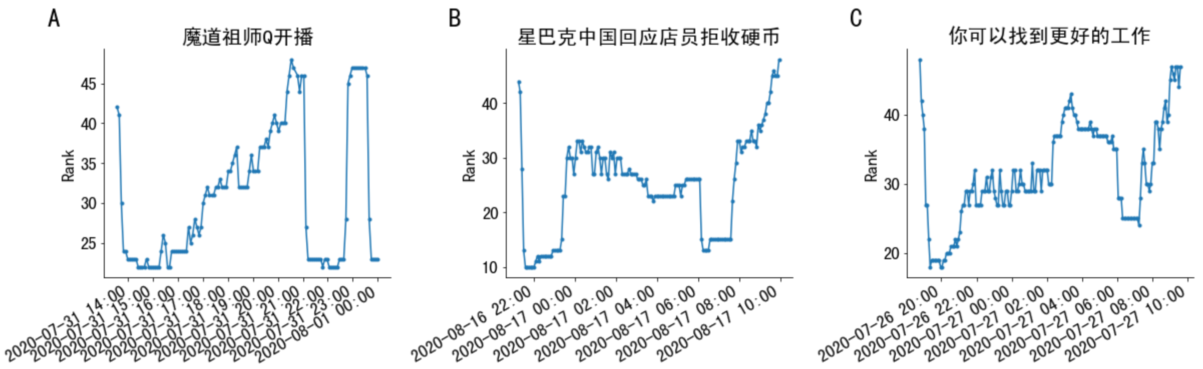

Figure S8. Examples of rank trajectories in Section 2 Cluster 3. (A) #Grandmaster of Demonic Cultivation Q start of broadcast# (#魔道祖师 Q 开播#) (B) (#Starbucks China responds to store clerk's refusal to accept coins#) #星巴克中国回应店员拒收硬币# (C) #You can find better jobs# (#你可以找到更好的工作#)

SI4. Rank trajectory clustering patterns with different number of clusters

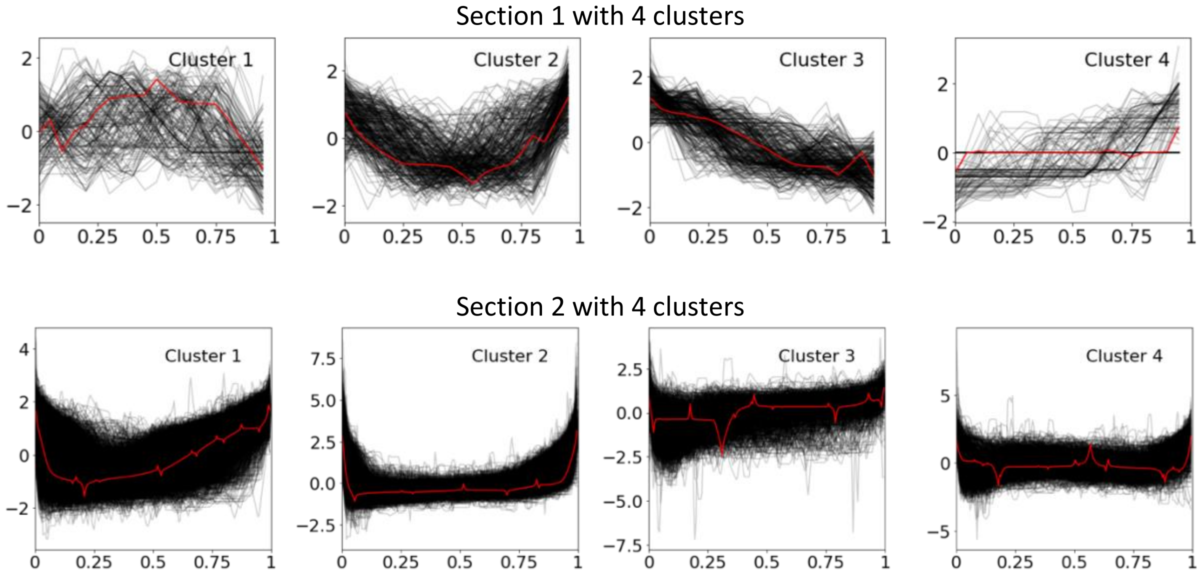

Figure S9. Rank trajectory clustering patterns of each section with different number of clusters.

SI5. Hashtags with high enter-rank and short duration

Table S2. Hashtags with high enter-ranks and short duration on Weibo HSL

Hashtags	Translation
令人叫绝的明星神颜	The amazing faces of the stars
这届妻子不好惹	Wives in this episode are not to be messed with
李斯丹妮汉服版无价之姐	Dany Lee Hanfu version of priceless sister
百家电影官微集体营业	Hundreds of official movie microblogs are open for business
明星跨界有多拼	How hard do stars cross the border
街舞 3 队长 battle	Street dance 3 captain battle
尹正太有梗了	Yin Zheng is so gag
潘晓婷台球教学	Pan Xiaoting billiards teaching
王者荣耀世冠神秘战队爆料	Honor of Kings world championship mystery team break the news
李荣浩套路终结者	Li Ronghao trick terminator
欧阳娜娜给薇娅采耳	Ouyang Nana picks Viya's ear
梁建章扮包拯带你游	Liang Jianzhang dressed as Bao Zheng to take you on a tour
云鹰飞将 QGFly 对线夫赖	Cloud Eagle Flying General QGFly vs. Fly
医生谢谢你	Doctor thank you
如何看出帅哥喜欢我	How to see that handsome guy likes me
女生玩游戏太上头了	Girls play games too addicted
陈一冰倒立涂口红	Chen Yibing upside down applies lipstick
健身素颜妆	fitness vegan makeup
秦昊宠女儿	Qin Hao spoils his daughter
和平精英 PEL 冠军	Game for peace PEL champion
足不出户云游王者	Remain within doors roam king
潮玩人类在哪里	Where are the tide playing humans
吴建豪灵魂拷问苏五口	Wu Jianhao soul searching Suwukou
雪梨苏芒谈年龄状态	Cherie Su Mang talks about age status
李汶翰壁咚张鹤伦	Li Wenhan kabe-don Zhang Lunhe
沈腾拒绝李汶翰	Shen Teng rejects Li Wenhan
被女玩家支配的恐惧	Fear of being dominated by female gamers
电竞为什么没有女职选手	Why there are no female professional players in e-sports
秦昊薇娅合唱	Qin Hao Weiya chorus
电子竞技莫得感情	e-sports got no feelings
雪梨杨天真合伙	Cherie Yang Tianzhen partnership

金晨郁可唯表情包	Jin Chen Yu Kewei image macro
镜头中的脱贫故事	The story of poverty eradication in the lens
TS 对阵 DYG	TS vs DYG

SI6. Hashtags leave HSL at rank 33

The following table shows the list of the hashtags that have left the re-ranked Sina Weibo Hot Search List (HSL) at the rank 33, together with their English translations. Among 177 such hashtags, 138 of them are related to news of regions and countries outside of mainland China as shown in bold, and an overwhelming number of them are related to international politics. Among those domestic hashtags, a large majority are related to domestic regulations or politics. Example rank trajectories are shown in Fig. S10.

Note: *the Weibo HSL we mention in this paper refers to the re-ranked HSL after removing the advertisements labeled with "Recommendation (荐)".*

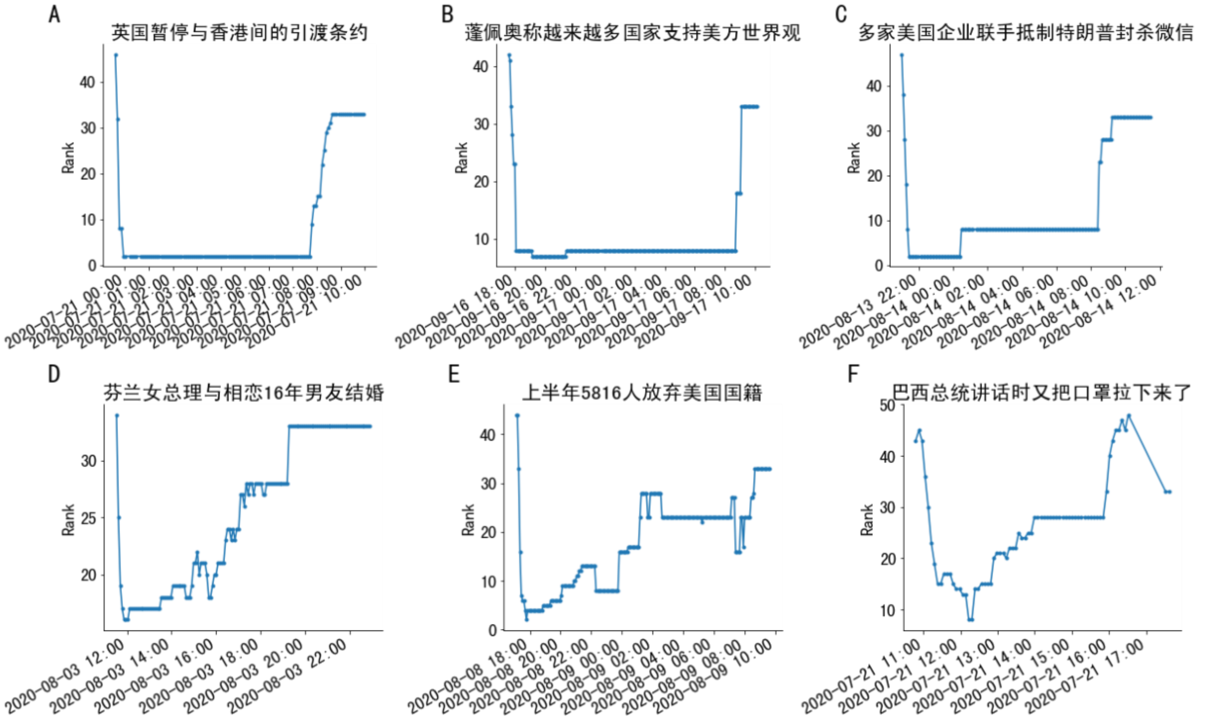

Figure S10. Examples of rank trajectories of hashtags leave HSL at rank 33 in the International category. Hashtags related to international politics have less fluctuations in their rank trajectories. (A) #UK suspends extradition treaty with Hong Kong# (#英国暂停与香港间的引渡条约#) (B) #Pompeo says more countries support US worldview# (#蓬佩奥称越来越多国家支持美方世界观#) (C) #Many U.S. companies join forces to resist Trump's blocking of WeChat# (#多家美国企业联手抵制特朗普封杀微信#) (D) #Finnish female PM marries boyfriend of 16 years in love# (#芬兰女总理与相恋 16 年男友结婚#) (E) #5,816 people renounce U.S. citizenship in first half year# (#上半年 5816 人放弃美国国籍#) (F) #Brazil's president pulled down the mask again while speaking# (#巴西总统讲话时又把口罩拉下来了#)

Table S3. List of hashtags that leave Weibo HSL at rank 33

Hashtag	Translation
31 省区市新增确诊 16 例	16 new cases confirmed in 31 provinces and cities
长征五号火箭垂直转运至发射区	Long March 5 rocket vertical transfer to the launch area
**扎克伯格连线福奇**	**Zuckerberg connected to Fauci**
国务院联防联控机制联络组离鄂返京	The State Council joint prevention and control mechanism liaison group left Hubei to Beijing
**特朗普称将恢复白宫每日疫情简报会**	**Trump says he will resume daily White House outbreak briefings**
**巴西总统讲话时又把口罩拉下来了**	**Brazil's president pulled down the mask again while speaking**
**外交部回应在永兴岛部署战斗机**	**Foreign Ministry responds to deployment of fighter jets in Yongxing Island**
**世卫反驳称蓬佩奥指责毫无根据**	**WHO counters that Pompeo's accusations are baseless**
**白俄总统确诊感染新冠病毒**	**Belarus president diagnosed with coronavirus**
**佩洛西要求美国会众议员佩戴口罩**	**Pelosi asks U.S. congressmen to wear masks**
科技部明确论文数量不可与奖励挂钩	Ministry of Science and Technology clarifies that the number of papers cannot be linked to awards
**外交部回应美国威胁巴西排斥华为**	**Foreign Ministry responds to US threat to reject Huawei in Brazil**
**国台办回应李登辉病亡**	**Taiwan Affairs Office responds to Lee Teng-hui's illness and death**
钟南山成为共和国勋章建议人选	Zhong Nanshan becomes recommended candidate for Order of the Republic
**外交部向 BBC 提出严正交涉**	**Foreign Ministry lodges stern representations with BBC**
**黎巴嫩环境部长辞职**	**Lebanon's environment minister resigns**
**港府全力支持中央政府对美反制**	**The Hong Kong government fully supports the central government's countermeasures against the US.**
**拜登提名美国副总统候选人**	**Biden nominates US vice presidential candidate**
**福奇质疑俄罗斯新冠疫苗**	**Fauci questions Russia's coronavirus vaccine**
测谎结果不属于合法证据形式	Polygraph results are not a legitimate form of evidence
商务部发文称将开展数字人民币试点	Ministry of Commerce issues document saying it will carry out digital yuan pilot
**外交部谴责美将孔子学院妖魔化污名化**	**Foreign Ministry condemns US demonization and stigmatization of Confucius Institute**
**香港警队回应英国禁止培训港警**	**Hong Kong police force responds to UK ban on training Hong Kong police**
**新西兰因疫情反弹决定推迟大选**	**New Zealand decides to postpone general election due to backlash against epidemic**

**外交部回应加拿大与 FBI 合谋搜查孟晚舟**	**Foreign Ministry responds to Canada's conspiracy with FBI to search Meng Wanzhou**
**商务部回应美封杀 TikTok**	**Commerce Ministry responds to US blocking TikTok**
**班农申请无罪审判**	**Bannon files for not guilty trial**
**班农交 500 万美元保释金离开法院**	**Bannon pays $5 million bail to leave court**
解放军和武警部队 120 多万人次抗洪	PLA and armed police forces more than 1.2 million people to fight floods
**全球已有超 8 万个新冠病毒基因序列**	**The world has more than 80,000 new coronavirus gene sequences**
**特朗普政府或允许美企在中国使用微信**	**Trump administration may allow U.S. companies to use WeChat in China**
**王毅批美国制裁守法国家荒唐透顶**	**Wang Yi criticizes US sanctions against law-abiding countries as absurd**
**驻以色列使馆提醒中国公民防范新冠**	**Embassy in Israel reminds Chinese citizens to guard against coronavirus**
**中方候选人当选国际海洋法法庭法官**	**Chinese candidate elected to International Tribunal for the Law of the Sea**
**巴西总统长子确诊感染新冠肺炎**	**Brazil president's eldest son diagnosed with coronavirus**
**联合国拒绝美要求恢复制裁伊朗**	**U.N. rejects U.S. demand to reinstate Iran sanctions**
**赵立坚回应美防长说准备对抗中国**	**Zhao Lijian responds to U.S. Defense Secretary's saying he's ready to confront China**
**美国国会将调查蓬佩奥**	**U.S. Congress to investigate Pompeo**
**默克尔称欧盟期待与中方进一步合作**	**Merkel says EU looks forward to further cooperation with China**
**德国柏林再现反防疫措施示威**	**Anti-epidemic measures demonstration in Berlin, Germany**
3 分钟混剪抗战史	3-Minute mix of war of resistance history
**特朗普公开攻击美军高层**	**Trump publicly attacks top US military**
**澳情报部门突击搜查中国驻澳记者住所**	**Australian intelligence raided the residence of Chinese journalists in Australia**
**94 岁英国女王将复工**	**Queen to return to work at age 94**
**水门事件调查记者揭露特朗普淡化疫情**	**Watergate investigative reporter exposes Trump's downplaying of epidemic**
**中国关于联合国成立 75 周年立场文件**	**China's position document on 75th anniversary of UN**
**外交部回应美正秘密发展新核武器系统**	**Foreign Ministry responds to US is secretly developing new nuclear weapons system**
**王毅称中国从不干涉美国内政**	**Wang Yi says China never interferes in US internal affairs**
廖国勋当选天津市市长	Liao Guoxun elected mayor of Tianjin
**中国驻英大使接受 BBC 专访**	**China's ambassador to Britain interviewed by BBC**
**美国防长称希望年内访问中国**	**U.S. Defense Secretary says he hopes to visit China within the year**

我国资源三号 03 星发射成功	China's Resource III 03 star launched successfully
北斗三号系统最后一颗组网卫星入网	The last satellite of the Beidou-3 system is on the network
**福奇称家人遭受死亡威胁**	**Fauci says family under death threats**
**刘鹤与美贸易代表通话**	**Liu He speaks with US trade representative**
**外交部批澳方无理搜查 4 名驻澳中国记者**	**Foreign Ministry criticizes Australia's unreasonable search of four Chinese journalists in Australia**
**特朗普对气变加剧山火表示怀疑**	**Trump expresses doubts about gas change exacerbating mountain fires**
**特朗普称发推后经常感到后悔**	**Trump says he often regrets after tweeting**
**美国总统国家安全事务助理感染新冠**	**U.S. Assistant to the President for National Security Affairs infected with coronavirus**
因疫情滞留新增食宿费用游客承担	Stranded due to the epidemic new accommodation and food costs borne by tourists
科技部要求严查违背科研诚信行为	Ministry of Science and Technology requires strict investigation of violations of scientific research integrity
**外交部回应蓬佩奥对李登辉表示哀悼**	**Foreign Ministry responds to Pompeo's condolences to Lee Teng-hui**
**外交部回应驻日美军成防疫体系漏洞**	**Foreign Ministry responds to the U.S. military in Japan has become a loophole in the epidemic prevention system**
**印度再禁 15 款中国 APP**	**India bans 15 more Chinese APPs**
人社部支持发展特色小店	The Ministry of Human Resources and Social Security supports the development of special small stores
张玉环回应申请国家赔偿	Zhang Yuhuan responds to application for state compensation
**崔天凯回应多个中美热点话题**	**Cui Tiankai responded to a number of China-US hot topics**
**俄罗斯副总理新冠阳性**	**Russian deputy prime minister's coronavirus positive**
**拜登首度携竞选搭档亮相**	**Biden's first appearance with his running mate**
新时代交通强国铁路先行规划纲要	The new era of strong transportation railroad first planning outline
杨克勤受贿案一审开庭	Yang Keqin's bribery case in the first trial
**近 7 成受访美企对中国市场前景乐观**	**Nearly 70% of surveyed U.S. companies optimistic about China market outlook**
**葡萄牙总统跳海救人**	**Portuguese president jumped into the sea to save people**
**安倍晋三进入庆应大学医院**	**Shinzo Abe enters Keio University Hospital**
**外交部回应特朗普再签涉 TikTok 行政令**	**Foreign Ministry responds to Trump signing another executive order involving TikTok**
**特朗普反击奥巴马夫人**	**Trump hits back at Mrs. Obama**

**香港将向世贸组织申诉**	**Hong Kong will appeal to the WTO**
**拜登成为美国民主党总统候选人**	**Biden becomes U.S. Democratic Party presidential candidate**
**英法德称美无权重启联合国对伊制裁**	**Britain, France and Germany say US has no right to reopen UN sanctions against Iran**
**外交部回应 TikTok 起诉美国政府**	**Foreign Ministry responds to TikTok's lawsuit against U.S. government**
**香港反对派议员林卓廷及许智峰被捕**	**Hong Kong opposition lawmakers Lam Cheuk-ting and Hui Chi-fung arrested**
**美国威斯康星州进入紧急状态**	**U.S. Wisconsin enters state of emergency**
**美国制裁研制新冠疫苗的俄罗斯机构**	**U.S. sanctions Russian agency that developed coronavirus vaccine**
**彭斯正式接受共和党副总统候选人提名**	**Pence formally accepts Republican nomination for vice president**
教育部将加强跟踪指导校园疫情防控	Education Ministry to strengthen follow-up on campus outbreak prevention and control
**国防部回应美拟在日部署中导**	**Defense Ministry responds to proposed US deployment of intermediate range missiles in Japan**
**毛里求斯爆发千人游行**	**Thousands march in Mauritius**
**菅义伟决定竞选自民党总裁**	**Yoshihide Suga decides to run for LDP president**
需要人陪	The need for company
**特朗普连发 4 推谴责波特兰示威**	**Trump condemns Portland protest in 4 tweets**
教育部发布高校命名最新规范	Ministry of Education releases latest norms for naming colleges and universities
**美国称不加入与世卫有关的疫苗开发**	**U.S. says it won't join WHO-related vaccine development**
**特朗普承认曾淡化新冠疫情严重性**	**Trump admits to having downplayed severity of coronavirus outbreak**
**中方要求美国不要干涉中国内政**	**China asks U.S. not to interfere in China's internal affairs**
**菅义伟当选日本自民党总裁**	**Yoshihide Suga elected president of Japan's Liberal Democratic Party**
**英国暂停与香港间的引渡条约**	**UK suspends extradition treaty with Hong Kong**
美国驻成都总领事馆现场情况	U.S. Consulate General in Chengdu on site
**王毅称中国将坚定而理性地回应美国**	**Wang Yi says China will respond firmly and rationally to U.S.**
**中方坚决反对人为制造所谓新冷战**	**China firmly opposes artificially created so-called new cold war**
全国危化品储存安全专项检查整治	National hazardous chemical storage safety special inspection and rectification
**外交部回应中加关系遭遇困难**	**Foreign Ministry responds to difficulties in China-Canada relations**
**普京宣布俄首个新冠疫苗注册**	**Putin announces registration of Russia's first coronavirus vaccine**
**特朗普遭 52 家科技巨头联名起诉**	**Trump was jointly sued by 52 tech giants**

检方通报李心草溺亡案件	The prosecution informed Li Xincao drowning case
**多家美国企业联手抵制特朗普封杀微信**	**Many U.S. companies join forces to resist Trump's blocking of WeChat**
**中国驻休斯敦总领事馆全体馆员归国**	**Chinese Consulate General in Houston returns all members to China**
**外交部回应美方宣布取消中美经贸谈判**	**Foreign Ministry responds to U.S. announcement to cancel U.S.-China economic and trade talks**
**特朗普呼吁抵制固特异轮胎**	**Trump calls for boycott of Goodyear tires**
**拜登称如果当选将要求全美戴口罩**	**Biden says he will ask all Americans to wear masks if elected**
**特朗普 83 岁姐姐录音**	**Trump's 83-year-old sister recording**
**特朗普被正式提名为共和党总统候选人**	**Trump was officially nominated as the Republican presidential candidate**
**国防部回应美军机擅闯我演习禁飞区**	**The Ministry of Defense responded to the U.S. military aircraft trespassed exercise no-fly zone**
**美宣布制裁 24 家参与南海建岛中企**	**The United States announced sanctions against 24 Chinese companies involved in the construction of islands in the South China Sea**
**安倍称辞职如断肠之痛**	**Abe says resignation like a broken heart**
**外交部回应向南海发射导弹报道**	**Foreign Ministry responds to reports of missile launches into South China Sea**
**日本政府计划 9 月 17 日选出新首相**	**Japanese government plans to elect new prime minister on Sept. 17**
**外交部副部长约见捷克驻华大使**	**Vice Foreign Minister meets with Czech Ambassador to China**
**王毅点名警告捷克参议长你过线了**	**Wang Yi warns Czech Senate President by name crossed the line**
**印度宣布再禁用 118 款中国 App**	**India announces ban on 118 more Chinese apps**
**印巴交火一名印度军官死亡**	**India, Pakistan exchange fire, one Indian officer dead**
**英国拟向欧盟发通牒**	**UK to issue ultimatum to EU**
**外交部驳斥美称中国操纵湄公河水资源**	**Foreign Ministry refutes US claim that China manipulates Mekong water resources**
**阿富汗副总统遇袭受轻伤**	**Afghan vice president slightly wounded in attack**
**中俄发布外交部长联合声明**	**China, Russia issue joint statement by foreign ministers**
**蓬佩奥称越来越多国家支持美方世界观**	**Pompeo says more countries support US worldview**
**日本新首相菅义伟首次记者会**	**Japan's New Prime Minister Yoshihide Suga's First Press Conference**
外交部新任发言人汪文斌	New Foreign Ministry spokesman Wang Wenbin
中组部划拨 1.2 亿元党费用于防汛救灾	Ministry allocates 120 million yuan in party expenses for flood relief

**外交部回应英国将暂停与香港引渡条约**	**Foreign Ministry responds to UK to suspend extradition treaty with Hong Kong**
坚决防止家长他人代劳等参赛造假行为	Resolutely prevent falsification of participation by parents and others on their behalf
**中方将考虑不承认 BNO 为有效旅行证件**	**China will consider not recognizing BNO as a valid travel document**
文化和旅游部副部长李金早被查	Vice Minister of Culture and Tourism Li Jinzao investigated
**芬兰女总理与相恋 16 年男友结婚**	**Finnish female PM marries boyfriend of 16 years in love**
刘永坦捐出最高科技奖 800 万奖金	Liu Yongtan donates 8 million prize money top science and technology award
**上半年 5816 人放弃美国国籍**	**5,816 people renounce U.S. citizenship in first half year**
**外交部说中国人民是吓不倒的**	**Foreign Ministry says Chinese people can't be intimidated**
十四五规划编制工作开展网上意见征求	The 14th Five-Year Plan preparation work to carry out online opinion solicitation
**王毅赴机场迎接驻休斯敦总领事馆馆员**	**Wang Yi Goes to Airport to Greet Consulate General in Houston**
**美国没资格要求安理会恢复对伊制裁**	**U.S. not qualified to ask Security Council to restore sanctions against Iran**
中国人民警察警旗式样	Chinese people's police flag style
**越南逮捕 21 名中国通缉犯**	**Vietnam arrests 21 wanted Chinese criminals**
**东部战区台海演练针对的就是台独**	**Eastern Zone of War's Taiwan Strait drills target Taiwan independence**
**安倍晋三就辞职举行记者会**	**Shinzo Abe holds press conference on resignation**
中央第七次西藏工作座谈会	The seventh central Tibetan work symposium
**特朗普说自己的签名值 1 万美元**	**Trump says his signature is worth $10,000**
**特朗普考虑限制中国留学生**	**Trump considers restricting Chinese students**
袁家军	Yuan Jiajun
**中印国防部长在莫斯科举行会晤**	**China, India defense ministers meet in Moscow**
**特朗普政府考虑将中芯国际列入黑名单**	**Trump Administration Considers Blacklisting SMIC**
**肯塔基州两支游行队伍现场对峙**	**Two Kentucky marches face off on site**
**美驻华大使布兰斯塔德将离任**	**U.S. Ambassador to China Branstad to resign**
王宁当选福建省省长	Wang Ning elected governor of Fujian province
**台湾新竹空军基地内直升机坠落**	**Taiwan's Hsinchu Air Force base helicopter crash**
**特朗普自夸疫情发布会收视率超高**	**Trump's boast of epidemic conference gets superb ratings**
**外交部披露美私拆中方外交邮袋细节**	**Foreign Ministry discloses details of U.S. private demolition of Chinese diplomatic pouch**

**中方回应美方强行进入驻休斯敦总领馆**	**China responds to U.S. forcible entry into Consulate General in Houston**
**印度将审核 275 款中国 APP**	**India to check 275 Chinese apps**
**杨洁篪谈中美关系**	**Yang Jiechi on China-US Relations**
国旗法国徽法迎来重要修改	National Flag Law and National Emblem Law See Important Amendments
**香港特区第六届立法会继续履行职责**	**HongKongSAR's 6th Legislative Council continues to perform its duties**
**美国考虑禁止疑似感染新冠公民回国**	**U.S. considers banning citizens suspected of infecting coronavirus from returning home**
**特朗普称哈里斯和拜登都是社会主义者**	**Trump says Harris and Biden are both socialists**
**特朗普考虑大选后撤换国防部长**	**Trump considers replacing defense secretary after election**
**白俄罗斯**	**Belarus**
**特朗普再次反对邮寄选票**	**Trump again opposes mail-in ballots**
第三次国家药品集中采购	The third national drug centralized procurement
**赵立坚双引号手势回应澳反华机构谣言**	**Zhao Lijian's double-quote gesture in response to rumors of anti-Chinese institutions in Australia**
广州市原常务副市长苏泽群被查	Former executive vice mayor of Guangzhou Su Zequn investigated
**俄外长称不会拒绝与中国开展 5G 合作**	**Russian foreign minister says won't reject 5G cooperation with China**
**安倍经济学 8 年回顾**	**Abe economics 8-year review**
**加拿大抗议者推倒首任总理雕像**	**Canadian protesters push down statue of first PM**
在线旅游网站不得大数据杀熟	Online travel sites must not big data-enabled price discrimination against existing customers
**外交部回应印度士兵在边界冲突中丧生**	**Foreign Ministry responds to Indian soldier killed in border clash**
**美方官员视中国留学生为间谍是妄想症**	**US officials see Chinese students as spies is paranoid**
被绑架的 17 头牦牛	17 yaks kidnapped
**对美加征关税商品第一次排除延期清单**	**First exclusion extension list of goods subject to tariff increase against US**
**外交部回应菅义伟当选日本首相**	**Foreign Ministry responds to Yoshihide Suga's election as Japanese PM**

References

[1] Center, S. W. D. 2015 weibo user development report. Weibo Report. https://data.weibo.com/report/reportDetail?id=333 (2016)

[2] GitHub repository https://github.com/cuihaosabrina/Sina_Weibo_Interventions
